# External Validation of the PLASE Score Prediction Model for PDA Surgery

**DOI:** 10.1007/s00246-025-04096-w

**Published:** 2025-11-13

**Authors:** Yusuke Morita, Satoshi Masutani, Kyongsun Pak, Tohru Kobayashi, Seiichi Tomotaki, Tetsuya Isayama, Katsuaki Toyoshima

**Affiliations:** 1 Department of Neonatology, Kanagawa Children’s Medical Center, Yokohama, Japan; 2 Department of Pediatrics, Saitama Medical Center, Saitama Medical University, Staff Office Building 110, 1981 Kamoda, Kawagoe, Saitama 350-8550 Japan; 3 Department of Data Science, Clinical Research Center, National Center for Child Health and Development, Setagaya, Japan; 4 Department of Pediatrics, Yokohama City University, Yokohama, Japan; 5 Department of Pediatrics, Graduate School of Medicine, Kyoto University, Kyoto, Japan; 6 Department of Neonatology, National Center for Child Health and Development, Setagaya, Japan

**Keywords:** Preterm, Patent ductus arteriosus, Ultrasound, Operation, Prediction

## Abstract

The PLASE score—a predictive model for subsequent patent ductus arteriosus (PDA) surgery using simple echocardiographic indices on day 3—has demonstrated strong predictive ability. To our knowledge, no predictive model for PDA surgery has undergone external validation. This study aimed to externally validate and update the PLASE score using an independent cohort. The PLASE score was applied to a validation dataset comprising infants admitted to Kanagawa Children’s Medical Center between 2019 and 2024 with a gestational age of 23 to 29 weeks. Discriminative performance was assessed using the area under the receiver operating characteristic curve (ROC-AUC) and the Brier score. Calibration analysis was also conducted. The PLASE score was subsequently updated using the entire dataset. The validation cohort included 127 patients—23 who underwent PDA surgery and 104 who did not. The PLASE score exhibited good discriminative ability, with a ROC-AUC of 0.833 (95% confidence interval [CI], 0.755–0.911) and a Brier score of 0.125. After model updating, the ROC-AUC improved to 0.846 (95% CI, 0.810–0.882). The PLASE score demonstrated high predictive performance in this external validation cohort. Based on the integrated dataset, a revised model (PLASE score version 2) was developed, offering a more robust and clinically applicable tool for current neonatal care.

## Introduction

Patent ductus arteriosus (PDA) is a major complication in preterm infants. Severe PDA is associated with increased mortality [1] and morbidities, including bronchopulmonary dysplasia [2], intraventricular hemorrhage [3], and necrotizing enterocolitis [4].Surgical ligation is generally considered for symptomatic PDA that is refractory to or contraindicated for medical treatment [5]. In preterm infants with hemodynamically significant PDA [6], cyclooxygenase (COX) inhibitors can be used to promote closure; however, their use carries a risk of complications and uncertainty in efficacy. Although even hemodynamically significant PDA may close spontaneously without treatment, timely administration of COX inhibitors is important when needed. Early prediction of the future need for PDA surgery may optimize circulatory management, including the use of COX inhibitors, in preterm infants.

Recently, a predictive model for PDA surgery was reported based on three simple echocardiographic indices measured at 3 days of age [7]. Using a database from the prospective, multicenter observational Patent Ductus Arteriosus and Left Atrial Size Evaluation (PLASE) study, conducted across 34 neonatal intensive care units (NICUs) in Japan [8], a predictive score—the PLASE score—was developed with high predictive ability of the future need for PDA surgery. Unlike previous PDA severity scores [9–11], the PLASE score was developed from a sufficiently large sample size, validated through cross-validation, and is notable in that it does not require organ blood flow or measurements tissue Doppler. Rather, it is calculated using gestational age and three simple echocardiographic indices obtained on day 3 [7]. However, the utility of the PLASE score has not yet been validated in an external cohort. This study aimed to externally validate and update the PLASE score using a database of preterm infants admitted to the NICU at Kanagawa Children’s Medical Center (KCMC) following the original PLASE study period.

## Methods

### Study Design and Patients

This study involved an external validation of the PLASE score using a retrospective, single-center cohort (the validation dataset) comprising patients admitted to KCMC from April 2019 to March 2024. The characteristics of the validation dataset were compared with those of the original PLASE study cohort, which included 710 preterm infants admitted to 34 NICUs across Japan between October 2015 and December 2016 [8].

Inclusion criteria for both cohorts were as follows [8]: (1) gestational age at birth between 23 weeks 0 days and 29 weeks 6 days; (2) admission to a participating NICU within 24 h after birth; and (3) survival beyond 24 h after birth. Exclusion criteria for both cohorts were: (1) chromosomal abnormalities; (2) multiple anomalies, apparent clinical syndromes, or congenital anomalies requiring surgery during infancy; (3) congenital heart disease other than PDA, patent foramen ovale, or persistent left superior vena cava; (4) congenital metabolic, endocrine, neuromuscular, or systemic bone disease; (5) family history of severe hereditary disorders such as neuromuscular disease or cardiomyopathy; and (6) critical conditions deemed ineligible for the study by attending physicians. Additionally, we excluded patients who underwent PDA surgery before echocardiography on day 3 and those with missing echocardiography data on day 3.

The decision to perform PDA surgery was based on the infant’s clinical condition, including hemodynamic instability, feeding difficulties, or worsening renal or respiratory function, and was ultimately made by the attending physician [12].

This study was approved by the institutional review boards of Kanagawa Children’s Medical Center (168-9) and Saitama Medical Center (TAKIKAN2024-037).

### Demographics and Clinical Data

We collected data on gestational age, birth weight, height, Apgar scores at 1 and 5 min after birth, small-for-gestational-age status, type of pregnancy (singleton or multiple), sex, place of birth, mode of delivery, number of antenatal steroid doses, use of prophylactic indomethacin, number of pharmacological treatments with COX inhibitors, and surgical closure of PDA.

### Echocardiographic Measurements

We also collected data on the following echocardiographic parameters on day 3 of life: left atrial volume, ductal diameter (PDAd), left ventricular end-diastolic diameter, left pulmonary artery end-diastolic velocity (LPAedv), and the ratio of left atrium to aortic root (LA/Ao). All echocardiographic examinations were performed by a single experienced neonatologist who had passed the quality control test for echocardiographic measurements [8], as part of routine clinical practice.

### Statistical Analysis

Data are summarized as mean ± standard deviation and stratified by PDA surgery status in both the PLASE and validation datasets.

Using echocardiographic findings and background variables from the PLASE study database, multiple logistic regression models were created, and the model with the maximum ROC-AUC was selected.

The PLASE score, *P* = 1/(1 + exp[-((10.7091) + (1.141)* PDAd + (0.036)* LPAedv + (0.6356)* LA/Ao + (−0.5652)*GA)]) [7], was applied to the validation dataset, and its discriminative performance was evaluated using the area under the receiver operating characteristic curve (ROC-AUC), with repeated 3-fold cross-validation (200 iterations). The 95% confidence interval (CI) was computed using the bootstrap method (1000 iterations). The Brier score was calculated to assess prediction error. Calibration was assessed using a calibration plot and the intraclass correlation coefficient (ICC).

Finally, the PLASE score was re-estimated using the combined PLASE and validation datasets, and the updated model’s odds ratios, 95% CIs, cross-validated ROC-AUC, and Brier score were reported.

All analyses were performed using R, version 4.1.2.

## Results

**Fig. 1:**
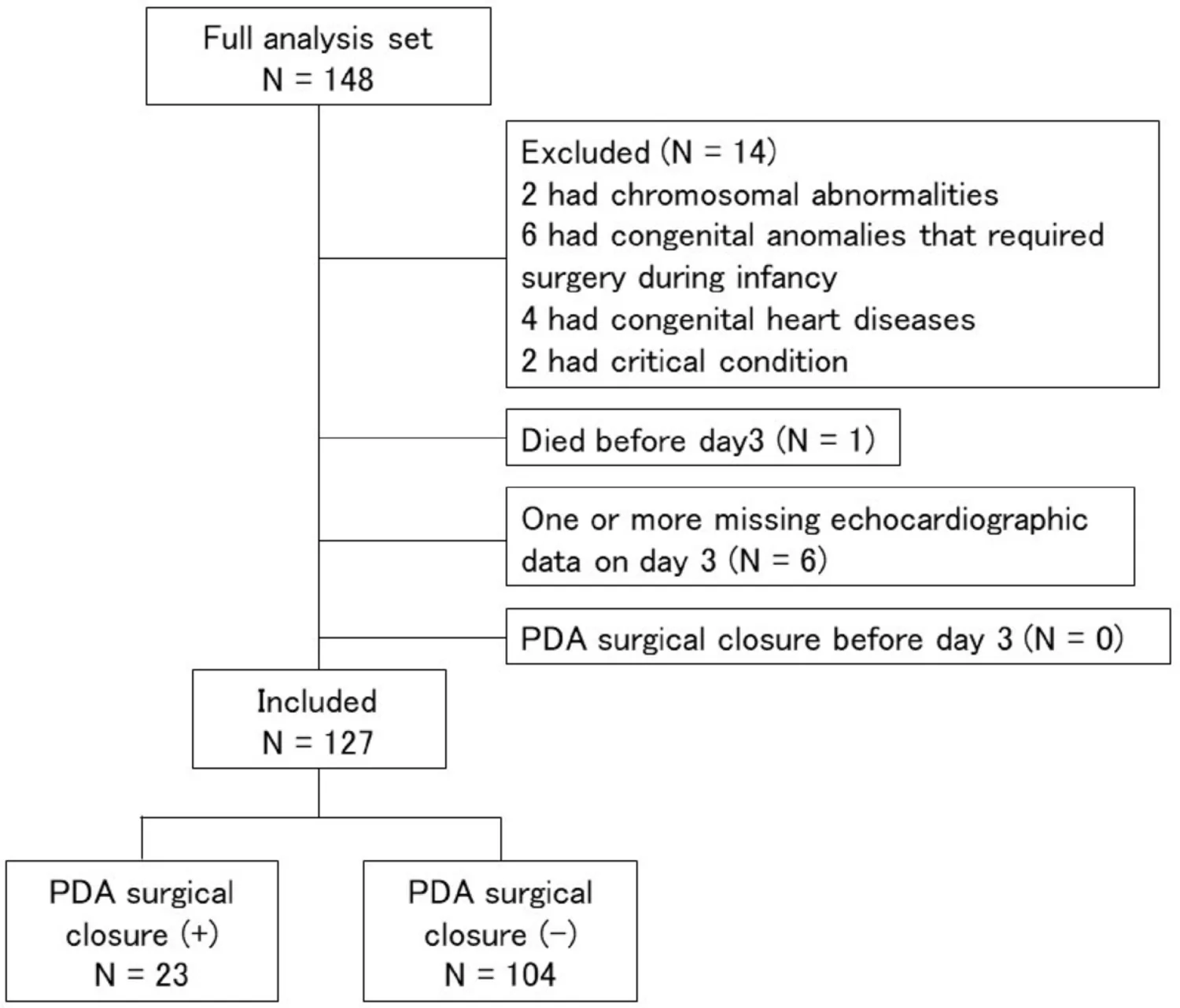
Flowchart of the study. A total of 148 preterm infants were initially assessed. Fourteen infants with congenital diseases or other exclusion criteria were removed. One infant died before day 3 of life, and six were excluded due to missing echocardiographic data on day 3. No infant underwent PDA surgical closure before day 3. Ultimately, 127 infants were included in the analysis, of whom 23 (18%) underwent PDA surgical closure. *PDA* patent ductus arteriosus

As shown in Fig. 1, among the 148 patients in the KCMC cohort, several were excluded based on the predefined criteria. Ultimately, 127 patients (23 with PDA surgical closure and 104 without) were included in the validation dataset.

Demographic and echocardiographic indices from both the PLASE and validation datasets are summarized in Table 1. The mean gestational age was 26.4 ± 1.9 and 26.3 ± 2.0 weeks, respectively, and the mean birth weight was 863 ± 262 and 863 ± 268 g, respectively. In the PLASE cohort, 77 of 692 patients (11.1%) underwent PDA surgery, whereas in the validation cohort, 23 of 127 patients (18.1%) underwent PDA surgery. The ages at PDA surgery were nearly identical—20 days and 19 days, respectively.

**Table 1:** Characteristics of patients

	PLASE dataset			Validation dataset		
	All	PDA surgical closure	No PDA surgical closure	All	PDA surgical closure	No PDA surgical closure
Demographics						
Number of patients	692	77	615	127	23	104
Gestational age (weeks)	26.4 ± 1.9	25.1 ± 1.7	26.5 ± 1.8	26.3 ± 2.0	24.6 ± 1.5	26.7 ± 1.9
Birth weight (g)	863 ± 262	740 ± 175	878 ± 267	863 ± 268	726 ± 217	894 ± 270
Birth height (cm)	33.1 ± 3.5	31.3 ± 2.7	33.3 ± 3.5	32.6 ± 3.4	30.8 ± 2.9	33.0 ± 3.3
Small for gestational age	156 (22.5%)	12 (15.6%)	144 (23.4%)	25 (19.7%)	1 (4.3%)	24 (23.1%)
Singleton (%)	560 (80.9%)	56 (72.7%)	504 (82.0%)	99 (78.0%)	19 (82.6%)	80 (76.9%)
Male sex (%)	377 (54.5%)	35 (45.5%)	342 (55.6%)	60 (47.2%)	12 (52.2%)	48 (46.2%)
In-hospital birth (%)	680 (98.3%)	76 (98.7%)	604 (98.2%)	120 (94.5%)	21 (91.3%)	99 (95.2%)
Cesarean section (%)	575 (83.1%)	68 (88.3%)	507 (82.4%)	111 (87.4%)	22 (95.7%)	89 (85.6%)
No antenatal steroid (%)	167 (24.1%)	17 (22.1%)	150 (24.4%)	21 (16.5%)	5 (21.7%)	16 (15.4%)
Apgar score, 1 min	4.1 ± 2.1	3.7 ± 2.1	4.2 ± 2.1	4.3 ± 2.0	4.2 ± 1.8	4.3 ± 2.1
Apgar score, 5 min	6.5 ± 2.0	6.2 ± 1.8	6.6 ± 2.0	6.9 ± 1.7	7.1 ± 1.7	6.9 ± 1.8
Prophylactic indomethacin (%)	218 (31.5%)	33 (42.9%)	185 (30.1%)	0 (0.0%)	0 (0.0%)	0 (0.0%)
Therapeutic cyclooxygenase inhibitor (%)	371 (53.6%)	71 (92.2%)	300 (48.8%)	73 (57.5%)	22 (95.7%)	51 (49.0%)
Echocardiographic indices at day 3						
LAV (mL)	0.493 ± 0.272	0.507 ± 0.331	0.491 ± 0.264	0.745 ± 0.238	0.759 ± 0.289	0.742 ± 0.227
PDAd (mm)	0.37 ± 0.63	0.94 ± 0.92	0.30 ± 0.55	0.62 ± 0.74	0.96 ± 0.72	0.55 ± 0.73
LVDd (mm)	11.7 ± 1.9	11.3 ± 2.1	11.8 ± 1.9	11.6 ± 2.0	10.4 ± 2.8	11.9 ± 1.7
LPAedv (cm/s)	5.9 ± 8.7	12.7 ± 11.1	5.0 ± 7.9	10.2 ± 6.4	10.5 ± 5.9	10.1 ± 6.6
LA/Ao	1.22 ± 0.21	1.32 ± 0.24	1.21 ± 0.20	1.25 ± 0.18	1.25 ± 0.22	1.25 ± 0.17
LAV (mL)/BBW (kg)	0.579 ± 0.271	0.669 ± 0.323	0.568 ± 0.262	1.189 ± 2.903	1.104 ± 0.507	1.207 ± 3.202
PDAd (mm)/BBW (kg)	0.45 ± 0.80	1.25 ± 1.19	0.35 ± 0.67	1.07 ± 3.38	1.27 ± 0.90	1.02 ± 3.71
LVDd (mm)/BBW (kg)	14.4 ± 3.4	15.6 ± 2.9	14.3 ± 3.5	17.3 ± 35.5	15.1 ± 3.9	17.8 ± 39.2
Outcome related variable						
PDA surgery performed (day)	–	20 ± 11	–	–	19 ± 8	–
Median [IQR]	–	21 [12–28]	–	–	20 [14–26]	–
[Min, Max]	–	[3, 45]	–	–	[6, 32]	–

*LAV* left atrial volume, *PDAd* patent ductus arteriosus diameter, *LVDd* left ventricular diastolic diameter, *LPAedv* left pulmonary artery end-diastolic velocity, *LA/Ao* left atrium to aortic root

**Fig. 2:**
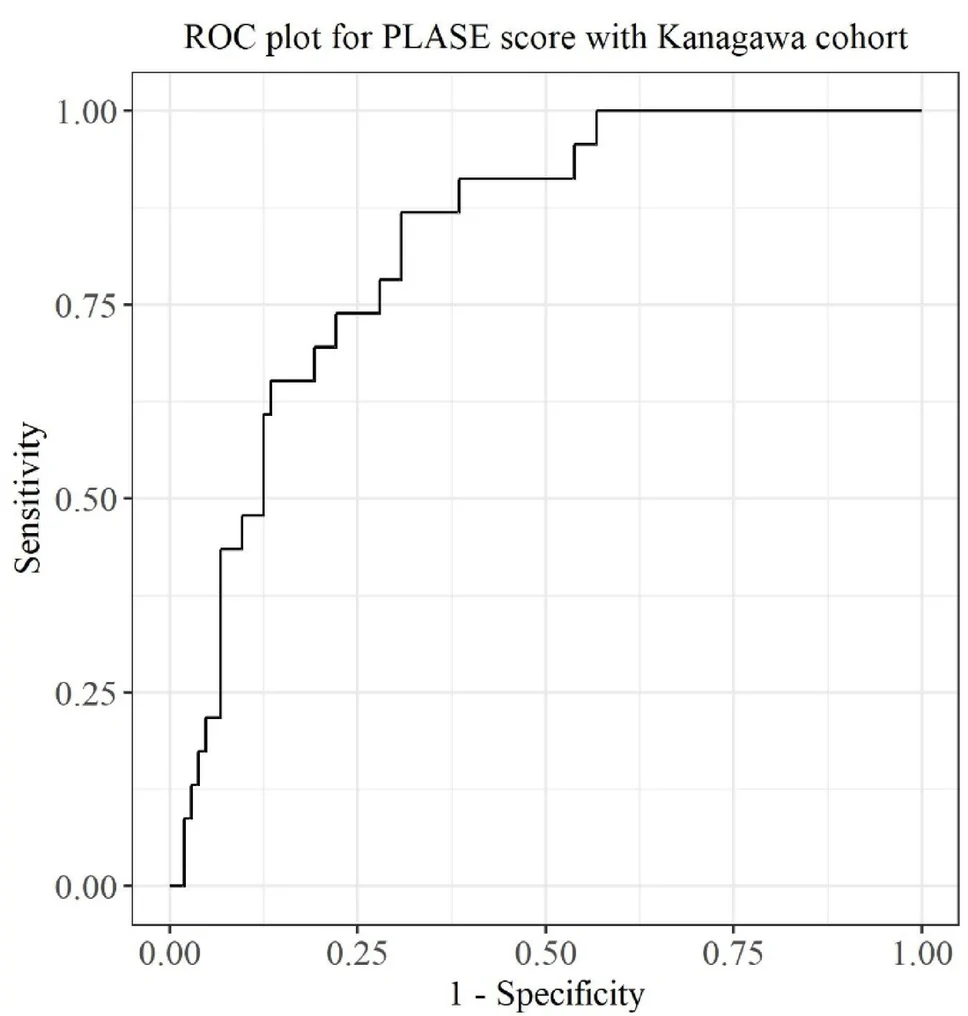
ROC curve for the prediction of PDA surgical closure using the PLASE score in the validation cohort. The PLASE score demonstrated good predictive ability, with an ROC-AUC of 0.833. *ROC* receiver operating characteristic, *PDA* patent ductus arteriosus, *AUC* area under the ROC curve

**Fig. 3:**
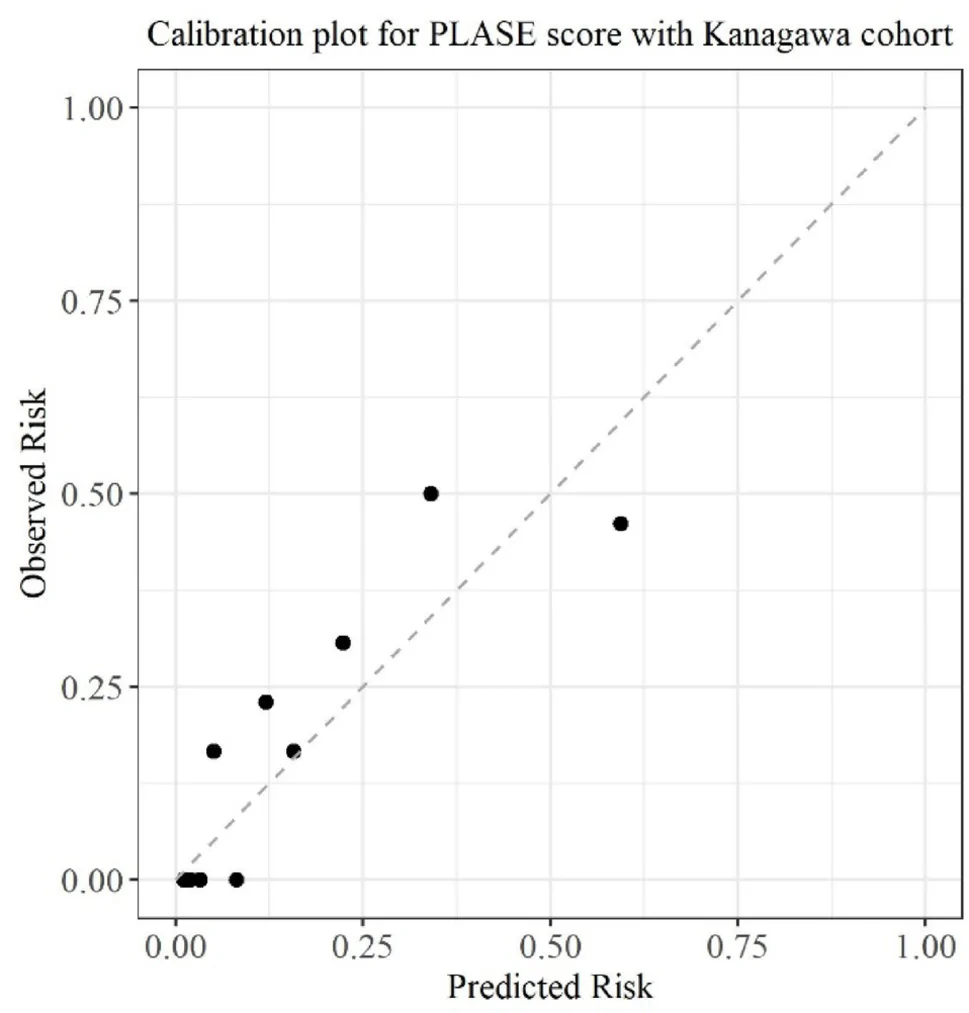
Calibration plot for the PLASE score in the validation cohort. The observed probabilities closely followed the ideal reference line (y = x), indicating strong agreement between predicted and actual probabilities of PDA surgical closure

The PLASE score developed from the PLASE dataset [7] was applied to the validation cohort (Table 2). In the original PLASE dataset, the score demonstrated good predictive ability with an ROC-AUC of 0.846 (95% CI, 0.805–0.886), a Brier score of 0.079, and an ICC of 0.949 (95% CI, 0.821–0.987) [7]. In the validation dataset, the PLASE score also demonstrated strong performance, with an ROC-AUC of 0.833 (95% CI, 0.755–0.911; Fig. 2) and a Brier score of 0.125. As shown in Fig. 3, the calibration plot for the validation dataset, along with an ICC of 0.880 (95% CI, 0.611–0.968), indicated satisfactory model fit. The observed data aligned well with the ideal reference line, demonstrating a strong agreement between predicted and observed probabilities of future PDA surgery.

**Fig. 4:**
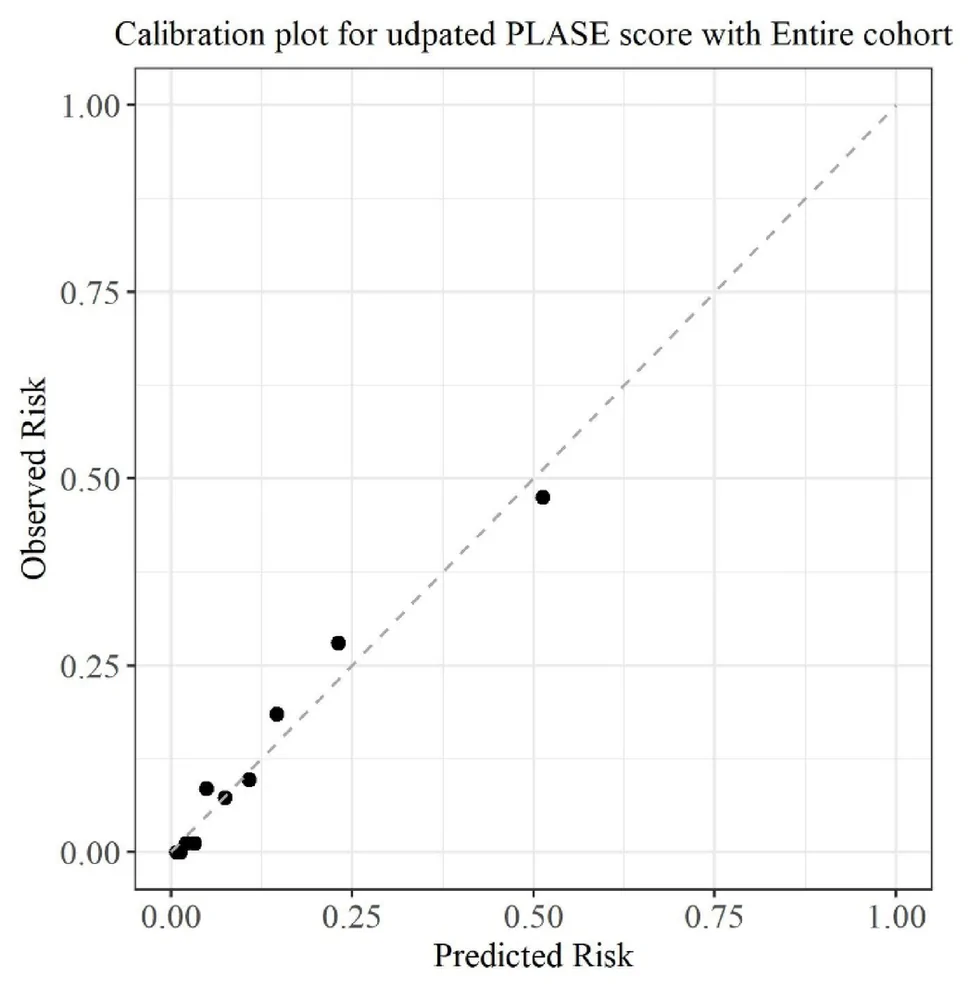
Calibration plot for the updated PLASE score (PLASE score version 2) in the entire cohort. The calibration curve closely approximated the ideal reference line (y = x), suggesting a strong fit between predicted and observed outcomes

Given the similarity between the PLASE and validation datasets, the two datasets were integrated, and the predictive model was re-estimated using the entire combined dataset. The updated model (PLASE score version 2) was defined as: *P* = 1/(1 + exp[−(12.5147 + 1.1608 × PDAd + 0.0289 × LPAedv + 0.2176 × LA/Ao − 0.6119 × GA)]). As shown in Table 3, the updated model demonstrated good predictive performance, with an ROC-AUC of 0.846 (95% CI, 0.810–0.882) and a Brier score of 0.086. The calibration plot for internal validation indicated excellent agreement, with an ICC of 0.984 (95% CI, 0.941–0.996; Fig. 4).

**Table 2:** ROC-AUCs and ICCs in validation dataset

	PDAd OR (95%CI), *p*-value	LPAedv OR (95%CI), *p*-value	LA/Ao OR (95%CI), *p*-value	Gestational age (weeks) OR (95%CI), *p*-value	ROC-AUC (95%CI)	Brier Score	ICC (95%CI), *p*-value
PLASE dataset	3.13 (1.98 – 4.95), <0.001	1.04 (1.01 – 1.07), 0.020	1.89 (0.53 – 6.74), 0.327	0.57 (0.48 – 0.67), <0.001	0.846 (0.805 – 0.886)	0.079	0.949 (0.821 – 0.987), < 0.001
Validation dataset					0.833 (0.755 – 0.911)	0.125	0.880 (0.611 – 0.968), < 0.001

*PDAd* patent ductus arteriosus diameter, *LPAedv* left pulmonary artery end-diastolic velocity, *LA/Ao* left atrium to aortic

**Table 3:** Updated ROC-AUC and ICC using PLASE and Kanagawa validation dataset

	PDAd OR (95%CI), *p*-value	LPAedv OR (95%CI), *p*-value	LA/Ao OR (95%CI), *p*-value	Gestational age (weeks) OR (95%CI), *p*-value	ROC-AUC (95%CI)	Brier Score	ICC (95%CI), *p*-value
PLASE + Validation dataset (Updated)	3.19 (2.12 – 4.82), < 0.001	1.03 (1.00 – 1.06), 0.055	1.24 (0.39 – 3.99), 0.714	0.54 (0.47 - 0.63), < 0.001	0.846 (0.810 – 0.882)	0.086	0.984 (0.941 – 0.996), < 0.001

Model	Probability Equation						
Updated PLASE PDA score	= 1/(1 + exp(-((12.5147) + (1.1608)*PDAd + (0.0289)*LPAedv + (0.2176)*LA/Ao + (−0.6119)*Gestational age)))						

## Discussion

This study represents the first external validation of the PLASE score and demonstrated its high predictive performance in an independent validation cohort. In addition, the predictive model was successfully updated using the combined dataset.

External validation of PDA prediction models and severity scores is clinically important. To date, Fink et al. applied the Shaare Zedek Medical Center (SZMC) score—graded from 1 to 3 (1: an insignificant PDA; 2: a borderline significant PDA; and 3: a hemodynamically significant PDA)—based on four echocardiographic parameters, to the cohort used for the El-Khuffash PDA severity score, which incorporates gestational age and four partially overlapping echocardiographic variables [9]. The El-Khuffash score increased with increasing SZMC grade [10]. Mullaly et al. reported that the El-Khuffash PDA severity score was significantly higher (i.e., worse) in treatment failure groups than in treatment success groups [13]. Although several PDA studies [14–16] have developed predictive models for severity or spontaneous closure, to our knowledge, none have been externally validated. In the present study, the PLASE score was applied to the KCMC cohort and exhibited strong predictive ability. The validation cohort differed from the original PLASE cohort in several respects. First, the validation data were collected more than three years after the PLASE study, during which time treatment strategies for PDA had evolved. Additionally, general neonatal care for preterm infants may have changed over that interval. Second, in the PLASE dataset, the criteria for PDA surgical treatment varied across centers, whereas at KCMC, surgical intervention is initiated relatively early once the patient is deemed suitable for surgery. Nevertheless, this study demonstrated similarly high predictive performance in the validation cohort. One possible reason is that background characteristics, such as gestational age and birth weight, were comparable between cohorts. Additionally, although the surgical criteria differed, the mean timing of surgery in the two datasets was similar. The PLASE score maintained its predictive performance across different datasets, supporting its generalizability.

The PLASE score is a simple tool that predicts the need for future surgery as early as day 3, using only three echocardiographic indices and gestational age. These indices are readily obtainable in routine clinical practice and are simpler than those included in other PDA severity scoring systems [9, 16]. Early identification of high-risk patients who may require future surgery is especially important in NICUs that need to transfer patients or coordinate with surgical teams for PDA closure.

### Study Limitations

This study has several limitations. First, there was variability in the decision-making processes for PDA surgery across NICUs. Some facilities, unlike KCMC, are unable to perform surgery on-site or experience substantial delays before surgery can be conducted. In such settings, the predictive ability of the PLASE score may differ, warranting future investigation. The validation dataset in this study was derived from a single-center cohort, and it remains unclear whether the PLASE score is applicable to institutions where surgical criteria differ significantly from those used in the original PLASE cohort.

Second, approximately 10 years have passed since the PLASE data were collected. During this time, therapeutic strategies for PDA and general neonatal care for preterm infants may have evolved. However, this study demonstrated that the PLASE score remained applicable to the validation cohort. Third, this validation cohort was derived from a single center in Japan. Therapeutic strategies for PDA may differ between Japan and other countries. For example, transcatheter occlusion of PDA in preterm infants has become increasingly common in other countries [17], but this procedure is available at only a few facilities in Japan. Therefore, the generalizability of the PLASE score to international settings remains uncertain.

## Conclusions

The PLASE score demonstrated high predictive ability in an external cohort. Based on the integrated dataset, a revised model (PLASE score version 2) was developed to offer a more robust and reliable tool for current clinical practice. However, further evaluation of its external validity will require future multicenter and international studies.

## Data Availability

The datasets generated and/or analyzed during the current study are available from the corresponding author upon reasonable request.
